# *CsCYP82D47* Is Identified as a Candidate Gene for Vivipary in Cucumber (*Cucumis sativus* L.)

**DOI:** 10.3390/ijms27167428

**Published:** 2026-08-19

**Authors:** Jingjing Xu, Tingting Fan, Yuxing Mo, Jintao Cai, Meina Liao, Zhaoyang Peng, Jing Zhou, Jing Zhao, Huiming Chen, Ruozhong Wang

**Affiliations:** 1 College of Bioscience and Biotechnology, Hunan Agricultural University, Changsha 410128, China; 13574368135@163.com (J.X.); ste0622@163.com (T.F.); 18607048937@163.com (J.C.); pengchaoyang@stu.hunau.edu.cn (Z.P.); zhoujing@hunau.edu.cn (J.Z.); zhj88717@live.vn (J.Z.); 2 Hunan Provincial Key Laboratory of the Traditional Chinese Medicine Agricultural Biogenomics, Changsha Medical University, Changsha 410219, China; 3 School of Life Science and Technology, Lingnan Normal University, Zhanjiang 524048, China; 18312738251@163.com; 4 Hunan Vegetable Research Institute, Hunan Academy of Agricultural Sciences, Changsha 410125, China; huiminghm@163.com

**Keywords:** cucumber, viviparous, BSA, QTL-seq, *CsCYP82D47*

## Abstract

Vivipary adversely affects the production process of the cucumber seed industry and greatly limits the popularization of cucumber varieties. Identification of the cucumber seed vivipary phenotype and screening of vivipary-associated genes will provide important theoretical value and practical significance for solving this problem in agricultural production. In this study, cucumber near-isogenic lines with significant differences in vivipary traits (viviparous line F and non-viviparous line BF) were successfully screened and used to construct genetic populations. Bulk segregant analysis (BSA) and QTL-seq were performed to fine-map the major-effect quantitative trait locus associated with vivipary variation. Based on QTL and BSA analyses, *CsaV3_3G044640* (designated *CsCYP82D47*), which encodes a cytochrome P450 family protein, was identified as a candidate gene associated with cucumber vivipary. *CsCYP82D47* exhibits obvious tissue specificity and is highly expressed in leaves, sprouts, and seeds. However, no significant difference in *CsCYP82D47* expression was detected between viviparous and non-viviparous cucumber materials. Further sequence analysis revealed multiple mutation sites in this gene between different genotypes. Specifically, the CYP82D47 protein in viviparous materials harbours one amino acid insertion (L63) and two missense mutations (M71L and S124L). In addition, the altered leucine residue distribution in viviparous cucumber may enlarge the substrate channel and enhance substrate catalytic efficiency, which may contribute to the vivipary phenotype. In summary, this study identifies a promising candidate gene potentially related to cucumber vivipary, which lays a foundation for further exploration of the molecular mechanism underlying cucumber vivipary and provides a potential genetic resource for cucumber molecular breeding.

## 1. Introduction

As one of the world’s top ten vegetables, cucumber (*Cucumis sativus* L.) is now widely planted in temperate and tropical regions, and it is the largest vegetable crop cultivated in the protected land in China [1]. Cucumbers, with their unique flavour and excellent characteristics as both vegetables and fruits, are becoming more and more popular, and the area under cultivation is still increasing. In the development of cucumber industry, the breeding of high-yielding and virus-resistant cucumber varieties has received widespread attention [2]. However, in the process of cucumber breeding, some cucumber varieties with excellent traits are not able to obtain high-quality seeds with industrial value due to seed viviparity at the breeding stage. These viviparous seeds have an extremely short dormancy period after maturity or continue to grow and develop in the mother without dormancy, which is termed viviparity, also known as preharvest sprouting (PHS) [3]. Viviparity occurs in many plants, and field crops such as wheat [4], rice [5], and corn [6], such as barley, are often referred to as ear sprouting. This is also the case with vegetables such as melons [7], tomatoes [8] and canola [9]. Currently, with the development of large-scale seed systems, the problem of viviparity has become increasingly prominent in reducing agricultural yields and has attracted global attention. Therefore, research on plant viviparity is growing rapidly, especially in traditional crops such as wheat, corn, and rice, and scientists are trying to explore effective technical means to solve this global challenge as soon as possible.

It is challenging to explore the gene regulation mechanism of seed dormancy and even preharvest germination. However, with the rapid development of molecular marker technology, Bulked segregant analysis sequencing (BSA-Seq) and Quantitative Trait Loci Mapping (QTL-seq) [10] methods came into being. BSA-Seq is a method that utilizes high-throughput sequencing techniques combined with mixed population isolation to rapidly pinpoint candidate intervals [11]. In this method, the individuals with the most viviparous and non-viviparous traits were first selected and mixed into two pools of phenotypic extremes, and then the two pools were resequenced. By comparing the variation frequency difference between the two extreme pools, the variation loci associated with the viviparous marker traits could be determined and the characters could be located. Bsa-based character mapping does not need to build genetic linkage map, which can speed up the identification process of gene loci and the breeding process.

A large number of scholars have mapped the dormant genes of rice seeds, providing a theoretical basis for finding the key genes controlling spike eruption. QTL analysis indicated that the dormancy/PHS characteristic of rice may be the result of co-determination of multiple genes. From the perspective of genetic factors, most researchers believe that PHS and dormancy of rice are quantitative traits jointly controlled by multiple genes, and the study of Zhao Fukuan et al. [12] on pumpkin fully shows that genotype is the main factor affecting the germination of seeds in melon. Cao Mingming et al. [13] found that the inheritance of the quantitative trait of viviparity in cucumber seeds is controlled by one pair of additive dominant major gene + additive dominant polygene. Based on this, studies on the number of genes specifically controlling preharvest germination traits and their regulatory mechanisms have been carried out successively. Currently, more than 80 QTLs related to PHS and dormancy have been detected on 12 chromosomes of rice [14]. Dong et al. [15] used a recombinant self-breeding population from Asominori/IR 24 to conduct a gene mapping study on seed PHS resistance. A total of six controlling panicle QTLs sites were detected by composite interval mapping, which were located on chromosomes 1, 4, 5, 7 and 8 respectively. Up to now, the genes related to PHS have expanded to 15, including *vP1*, *vP2*, *vP5*, *vP7*, *vP8*, *vP9*, *vP10*, *vP12*, *vP14*, *vp15, all*, *y9*, *w3*, *rea and dek33* [14,16,17,18,19,20]. Some scholars have also studied the resistance inheritance of preharvest germination traits. A few major loci have been consistently shown to account for a significant proportion of natural variation to sprouting in diverse mapping populations. Juthamas et al. [21] showed that PHS tolerance is controlled by additive major gene effects with minor gene effects where variations in minor gene effects were still unclear. Chang Cheng found that the QTLs located on chromosomes 3 and 4 played a significant role in PHS resistance of wheat [22].

A large body of research has demonstrated that plant endogenous phytohormones exert profound influences on seed development, dormancy and germination. Crosstalk among distinct hormones forms intricate regulatory networks that collectively govern seed developmental processes [23,24]. The antagonistic module formed by abscisic acid (ABA) and gibberellin (GA) acts as the core regulatory pathway underlying preharvest sprouting (vivipary). A series of pivotal functional genes controlling seed dormancy and germination have been characterized in horticultural crops. Among cucumber regulatory components, the CsABI3 and Cs*BPC* gene families serve as master modulators of seed dormancy [25]. The cucumber Cs*BPC* transcription factor regulates seed dormancy and germination via transcriptional repression of CsABI3, which defines a unique regulatory cascade for seed developmental balance. Beyond gene characterization, CRISPR-Cas genome editing has been widely adopted to manipulate dormancy-associated hormone genes in multiple crops [26]. Targeted mutagenesis of ABA biosynthesis and signal transduction genes can drastically modify seed dormancy strength and resistance to vivipary, offering solid molecular evidence to dissect the genetic basis of preharvest sprouting and facilitate germplasm improvement. Classic three-factor theory of seed dormancy and germination elaborates the balanced functions of GA, ABA and cytokinin: GAs accelerate seed germination, ABA suppresses embryonic growth, and cytokinins exert dual effects by alleviating ABA-mediated growth inhibition [27]. Apart from these core hormones, salicylic acid boosts seed water uptake and weakens seed coat hardness to stimulate embryonic outgrowth [28], jasmonic acid positively modulates seed germination and root elongation [29], and indole-3-acetic acid (IAA) elevates enzymatic activity and metabolic rates within seeds to promote germination [30].

Nevertheless, existing studies on cucumber vivipary reveal two notable research gaps that the present work attempts to partially address. Most previous genetic analyses of cucumber preharvest sprouting (PHS) have only yielded preliminary QTL intervals with relatively low resolution, and few studies have further delimited these intervals down to individual functional candidate genes; to our knowledge, no core regulatory gene for cucumber vivipary has been cloned and functionally characterized so far. In addition, earlier investigations into cucumber vivipary were largely limited to phenotypic and genetic-segregation analyses. Multi-dimensional evidence combining cytological observation, endogenous hormone quantification, gene expression profiling, and protein structure simulation remains insufficient for dissecting its underlying molecular mechanism.

In the present study, we identified a candidate gene *CsaV3_3G044640* (designated Cs*CYP82D47*) encoding a cytochrome P450 protein that is potentially associated with cucumber vivipary. Natural sequence variations were detected between viviparous and non-viviparous cucumber lines. We hypothesize that these mutations remodel the protein substrate channel architecture, which may boost catalytic capacity and accelerate IAA biosynthesis, ultimately triggering in situ seed germination inside fruits. Our findings provide new clues to decipher the molecular basis of vivipary in cucumber and other cucurbit crops and lay a theoretical foundation for mitigating preharvest sprouting during cucumber seed production.

## 2. Results

### 2.1. Comparison of Phenotype and Viviparous Rate of Different Varieties of Mature Cucumber

**Figure 1:**
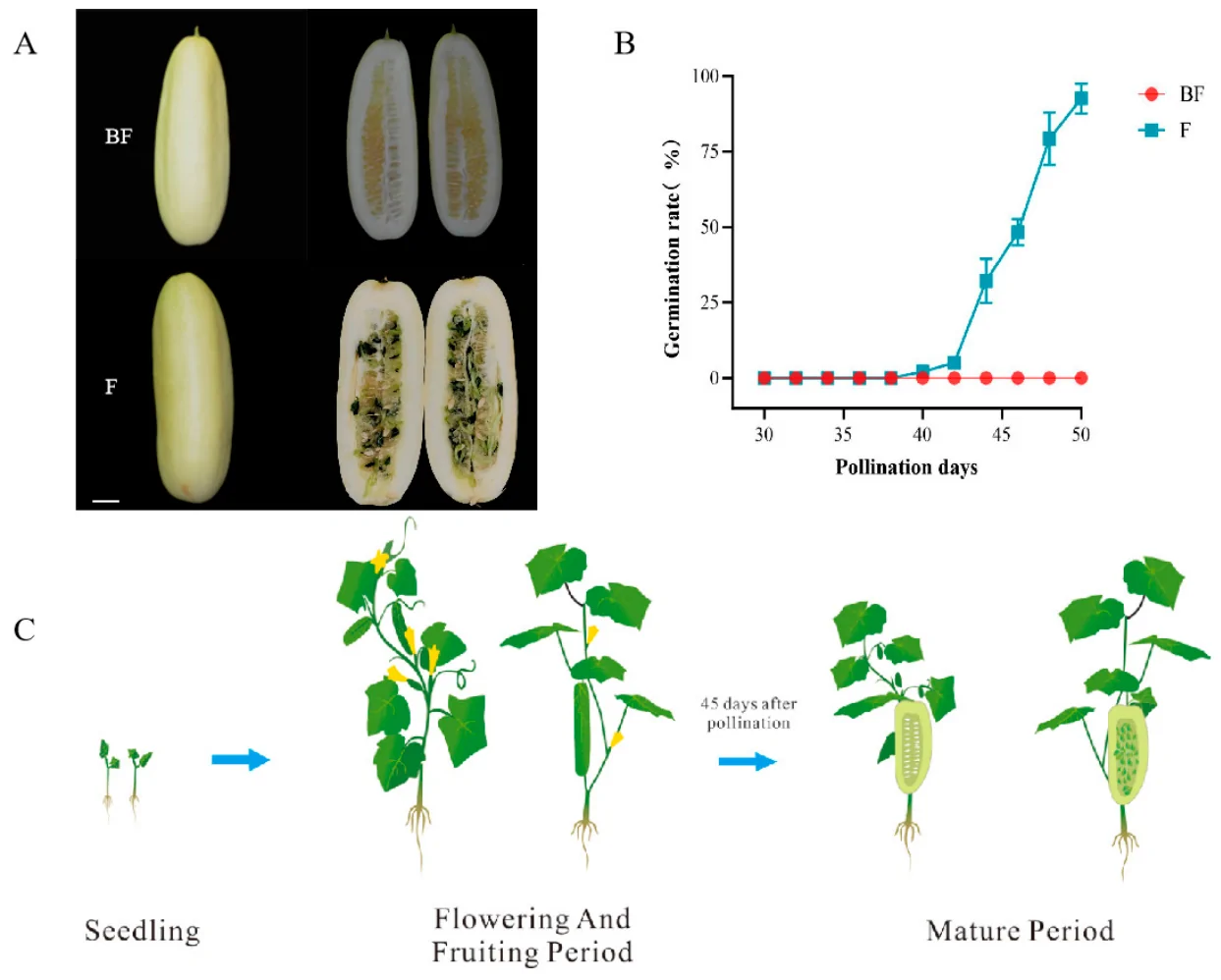
Comparison of phenotype and viviparity rate of different varieties of mature cucumber. (**A**): Longitudinal plot of different cucumber varieties pollinated for 45 days (scale is 3 cm; F: viviparous materials; BF: non-viviparous material; the same below.). (**B**): Comparison of differences in the rate of viviparity. (**C**): Growth phenotypes of different varieties of cucumber (all the cucumbers from seedling to maturity; (left) are BF (non-viviparous cucumbers) and (right) are F (viviparous cucumbers)).

The longitudinal slices of the cucumber species pollinated by F and BF for 45 days are shown in Figure 1A. As shown in the figure, the F cucumber seeds were fully generated in the fruit, the radicle was extended, the root whiskers grew and the tycotyl was exposed, the cotyledon stretched after the seed coating fell off, and the cotyledon turned green. After continuous planting, observation, and statistics, cucumber variety F, which is prone to vivipary, shows a vivipary rate of about 1.6% at approximately 40 days after pollination (Figure 1B). As time goes on, this phenomenon becomes more and more obvious; on the 44th day after pollination, the germination rate reached 30.5%. At 48 days after pollination, the viviparous rate climbed rapidly, reaching 87.8 percent, by which time most seeds had developed roots, and the cotyledon had begun to turn green. At 98.2% of the germination rate, except for the dented seeds, the rest of the seeds have germinated, two green cotyledon can be clearly seen, and can survive after transplantation. However, there was no sprout phenomenon in the process of seed production of cucumber BF. We plotted viviparous phenotypes from seedling to maturity for two cucumber species (Figure 1C).

### 2.2. Dynamics of Phytohormones During Seed Maturation of Two Cucumber Species and Comparison of Paraffin Sections of Seeds

**Figure 2:**
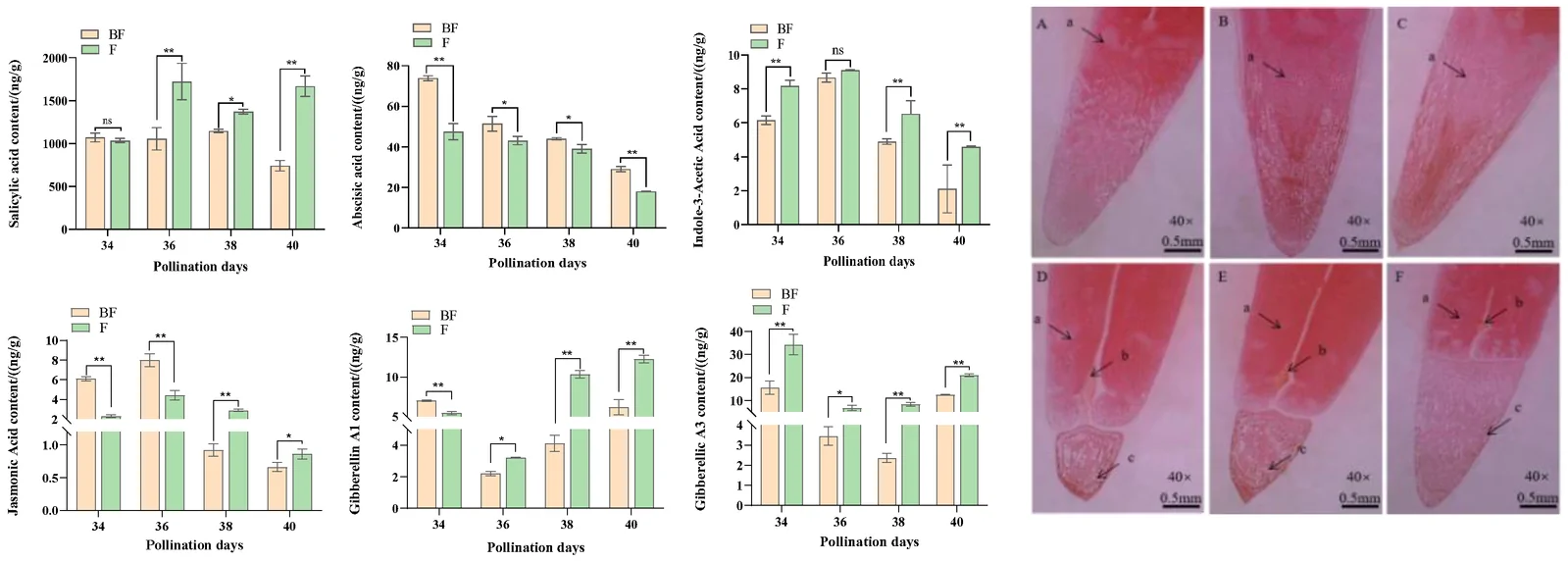
The dynamic changes in plant hormones during seed ripening in two kinds of cucumber fruits and longitudinal diagram of paraffin sections of seeds pollinated for 40 days. (**A**–**C**) Paraffin-section observations of seeds of line BF at 40 days after pollination; (**D**–**F**) Paraffin-section observations of seeds of line F at 40 days after pollination. Note: a: cotyledon; b: germ; c: radicle. * *p* < 0.05, ** *p* < 0.01.

After 30 days of pollination, the seeds of the cucumber gradually mature, and we will open the cucumbers pollinated for 34–40 days and take fresh seeds to measure the content of plant hormones. The total ion chromatogram (TIC), extracted ion chromatogram (XIC), and chromatograms of six plant hormones (SA, ABA, IAA, JA, GA1 and GA3) are displayed in Supplemental S1. As can be seen from Figure 2, at pollination day 34, the seeds in both cucumbers have become full, and the content of salicylic acid, abscisic acid, jasmonic acid, and gibberellin 1 in the non-germinated material BF seeds is higher than that in the germinated material F, while the content of indole-3-acetic acid and gibberellin 3 in the non-germinated material seeds is lower than that in the germinated material F. Firstly, the accumulation of salicylic acid in seeds is analyzed. At pollination day 34, there is no significant difference in salicylic acid content between the two materials, with the salicylic acid content in F being 1033.48 ng/g and the salicylic acid content in BF seeds being 1070.62 ng/g. At pollination day 36, the salicylic acid content in F seeds increases to 1722.76 ng/g, and F/BF is 1.63, with the salicylic acid content in BF seeds slightly decreasing to 1055.52 ng/g compared to two days ago. At pollination day 38, the salicylic acid content in F seeds slightly decreases compared to two days ago, but it is still higher than that in BF material. At pollination day 40, F material begins to show signs of germination, while BF seeds remain dormant. At this time, the salicylic acid content in F seeds is significantly higher than that in BF material, and F/BF is 2.25. In summary, during the period from pollination day 34 to 40, the salicylic acid content in F cucumber seeds presents a fluctuating pattern of rising–falling–rising, while the salicylic acid content in BF cucumber seeds presents a pattern of rising–falling. Secondly, we analyzed the accumulation of abscisic acid (ABA) content in seeds. At the pollination stage of 34–40 days, the ABA content in both types of cucumber fruits was found to decrease. At the pollination stage of 34 days, the ABA content in F was significantly lower than that in BF, with an ABA content of 47.52 ng/g in F and 73.87 ng/g in BF, with a ratio of 1.55. Over the next 6 days, the ABA content in F material continued to decrease to 18.04 ng/g, while the ABA content in BF material steadily decreased to 29.03 ng/g. At the pollination stage of 40 days, which was the germination node of F material, the ABA content in BF material was still higher than that in F material. Then, we analyzed the changes in indoleacetic acid (IAA) content in the seeds. Between 34 d and 36 d after pollination, the IAA content in both types of cucumber fruit increased and then decreased from 36 d to 40 d after pollination. At pollination 34 d, the IAA content in BF was significantly lower than that in F. At this time, the IAA content in BF was 6.15 ng/g, while the IAA content in F was 8.16 ng/g. At pollination 36 d, the IAA content in BF increased to 8.66 ng/g, and the IAA content in F increased to 9.09 ng/g. For the next 4 days, the IAA content in F material continued to decrease to 4.58 ng/g, while the indoleacetic acid content in BF material steadily decreased to 2.11 ng/g. At pollination 40 d, the IAA content in F material was significantly higher than that in BF material, and F/BF was 2.17. Next, we analyzed the changes in jasmonic acid content in the seeds. In the period from pollination day 34 to 36, the jasmonic acid content in both types of cucumber fruit increased and then decreased in the period from pollination day 36 to 40. The jasmonic acid content in both types of cucumber fruit decreased. At pollination day 34, the jasmonic acid content in BF was significantly higher than that in F. At this time, the jasmonic acid content in BF was 6.11 ng/g, while the jasmonic acid content in F was 2.28 ng/g, and the ratio of F to BF was 2.67. At pollination day 36, the jasmonic acid content in BF increased to 7.99 ng/g, and the jasmonic acid content in F increased to 4.41 ng/g. For the next 4 days, the jasmonic acid content in F material continued to decrease to 0.86 ng/g, while the jasmonic acid content in BF material steadily decreased to 0.66 ng/g. At pollination day 40, the jasmonic acid content in F material was significantly higher than that in BF material. Next, we analyzed changes in gibberellin content in seeds. At 34–36 d pollination, the gibberellin 1 in the two kinds of cucumber fruits showed a decreasing trend, and at 36–40 d pollination, the contents of gibberellin 1 in the two kinds of cucumber fruits showed an increasing trend. At 34 d pollination, the content of gibberellin 1 in BF was significantly higher than that in F. At this time, the content of gibberellin 1 in BF was 7.02 ng/g and the content of gibberellin 1 in F was 5.44 ng/g. On day 36 of pollination, the content of gibberellin 1 in BF and gibberellin 1 in F decreased to 2.19 ng/g and 3.21 ng/g respectively. After 38 days of pollination, the content of gibber 1 in F material was significantly higher than that in BF material, and the content of gibber 1 in BF material increased to 4.11 ng/g, and that in F material increased to 10.36 ng/g, and the F/BF was 2.52. After 40 days of pollination, the content of gibberellin 1 in F material was still significantly higher than that in BF material, and F/BF was 1.98. Finally, the change in gibberellin 3 content was analyzed. At 34–40 d of pollination, the gibberellin 3 in the two kinds of cucumber fruits decreased first and then increased, and the content of gibberellin 3 in F material was significantly higher than that in BF material. At 34 d pollination, the content of gibberellin 3 in BF was significantly lower than that in F. At this time, the content of gibberellin 3 in BF was 15.50 ng/g, the content of gibberellin 3 in F was 34.25 ng/g, and the F/BF was 2.21. On day 36 of pollination, the content of gibberellin 3 in BF and gibberellin 3 in F decreased to 3.44 ng/g and 6.68 ng/g respectively. After 38 days of pollination, the content of gibberellin 3 in BF material decreased to the lowest value of 2.36 ng/g, while the content of gibberellin 3 in F material increased to 8.29 ng/g and F/BF was 3.51. After 40 days of pollination, the content of gibberellin 3 in F material was 1.98 times higher than that in BF material.

After paraffin section examination (Figure 2), 40 days after pollination of F seed, the radicle protruding from the outside of the seed coat can be clearly seen (Figure 2D–F), which is due to the continuous growth of hypocotyl cells leading to the root end passing through the seed coat, while the germ and cotyl are scattered on both sides of the cotyledon. The morphological characteristics of dicotyledon are very obvious. The hypocotyledon is connected with the cotyledon, the germ has the first true leaf base and growth point, and the morphological characteristics of dicotyledon are very obvious. BF seed radicle did not extend, and no germ grew; the storage tissue, cotyledon (Figure 2A–C), occupied almost the whole seed, and endosperm nutrients are absorbed by the embryo, forming endosperm free seeds. It can be seen from Figure 2 that F seed has been in the germination state, while BF seed is still in the dormancy state.

### 2.3. Genetic Analysis of Viviparous Traits in Cucumber

**Figure 3:**
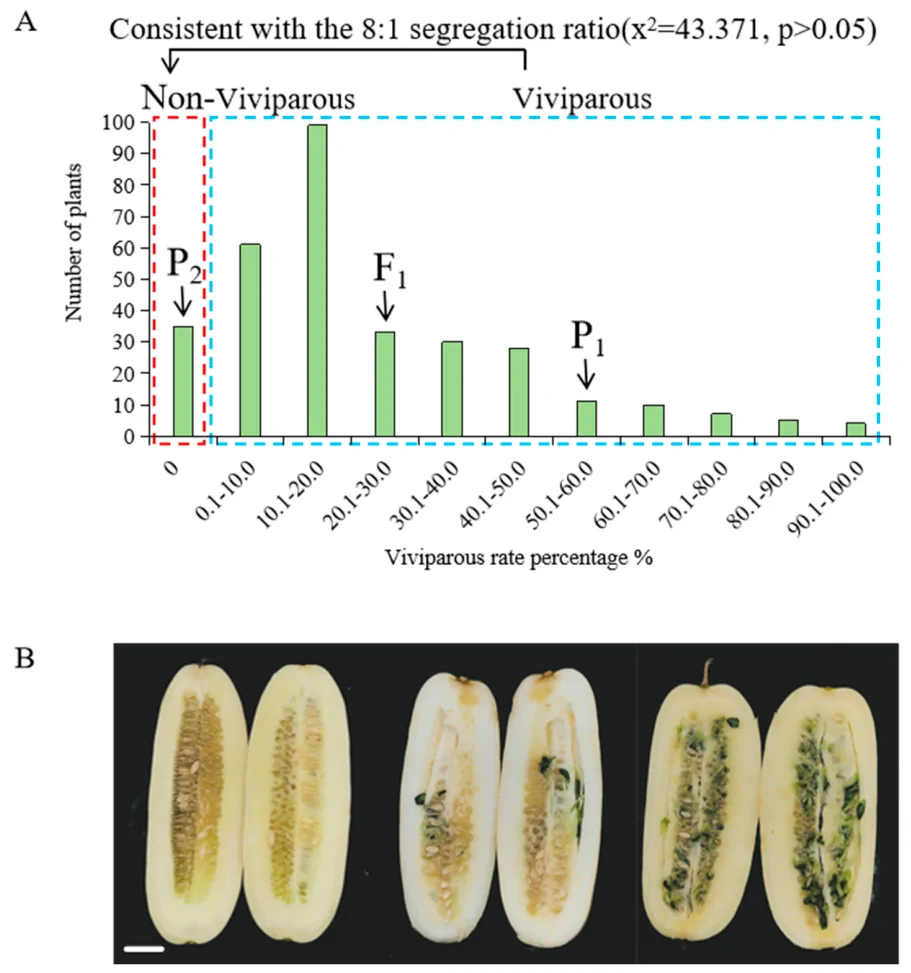
Frequency distribution of the percentage of viviparous rate (**A**) and phenotypic identification of F_2_ cucumber fruits (**B**). Note of (**A**): The red dashed border represents the frequency of non-viviparous cucumber seeds, and the green dashed border represents the frequency of viviparous cucumber seeds. Note of (**B**): No viviparous occurred (left), medium degree (middle), severe degree (right). Scale bar = 3 cm.

With stable genetic F (viviparous) as parent, the F_1_ and F_2_ generation populations were constructed by crossbreeding with BF (non-viviparous). A small number of seeds in F_1_ had the phenomenon of germination. F_2_ generation was obtained after F_1_ self-fertilization, and the characters of viviparous and non-viviparous separation appeared in F_2_ population. Fifty days after pollination in F_2_ generation, 377 cucumber seeds were sliced and dissected, and the results of germination of a single cucumber seed were counted, as shown in Table 1. The ratio of viviparity to non-viviparity of offspring was about 8:1. To determine whether the viviparous traits of cucumber fruits are controlled by single or multiple genes, we analyzed the frequency distribution of viviparous percentage in P_1_ (F), P_2_ (BF), F_1_ (F × BF), and F_2_ (Figure 3A). The results showed that the average percentage of parental P_1_ (F) was mainly between 50.1% and 60.0%. The non-viviparous material P_2_ (BF) has stable characteristics, and the vivipary rate is 0%. The average percentage of births in F_1_ was 20.1% and 30.0%. Isolated population F_2_ showed a single-peak left-biased normal distribution, with a single-peak distribution range from 10.1% to 20.0%, indicating that a major gene may be affected by the environment (Table S1 and Table S2).

### 2.4. Screening of Cucumber Viviparous Genes Based on BSA Whole-Genome Resequencing

To identify the causal mutations underlying the viviparous phenotype, bulked segregant analysis sequencing (BSA-seq) was performed using the F (viviparous) and BF (non-viviparous) lines. To guarantee high-quality sequencing data, raw sequencing data totaling 56.76 G were processed for quality filtering; 0.52 G of invalid reads were discarded, yielding 56.24 G of clean data. The raw data output for each sample ranged from 12,692.7 M to 15,047.711 M, exhibiting satisfactory sequencing quality (Q20 ≥ 96.29%, Q30 ≥ 91.13%), with GC content varying from 37.84% to 38.03%. The cucumber reference genome assembly used in this study has a total size of 226,211,662 bp. The mapping rate across all samples varied from 96.06% to 96.64%, the average genomic coverage depth ranged from 43.03× to 52.11×, and the 1×-coverage ratio exceeded 98.74%.

**Figure 4:**
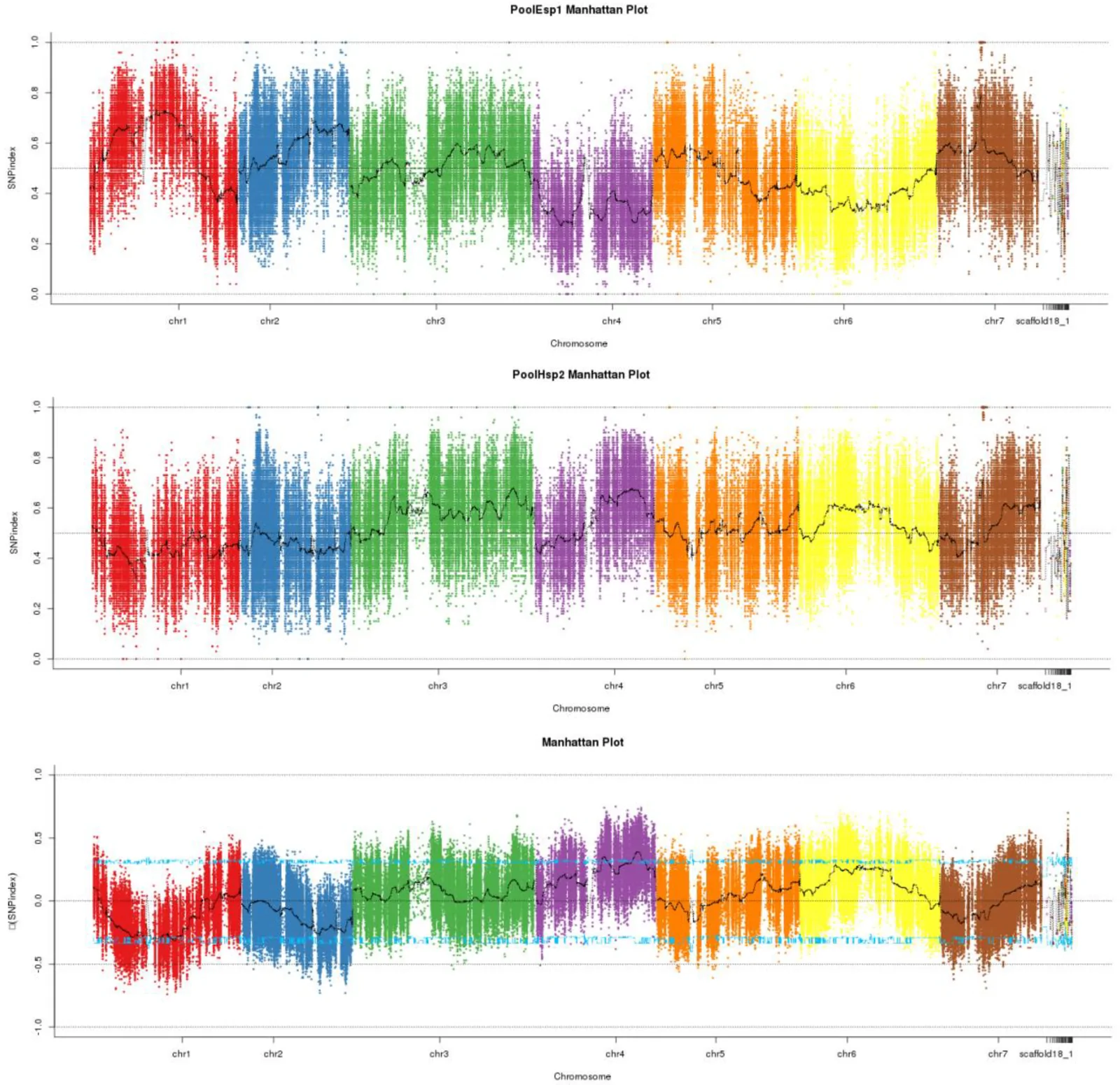
SNP/InDel-index Manhattan graphs of R-pool, S-pool and Δ (SNP/InDel-index) from QTL-seq approach for mapping the genomic regions controlling viviparity in cucumber.

Based on the genotyping information of the two parental lines, homozygous polymorphic markers were extracted, and a total of 384,679 polymorphic sites were initially identified. After SNP-index calculation, 377,001 polymorphic sites were further filtered. We retained markers that were homozygous and polymorphic between the two parents and removed sites with missing SNP-index values in the segregating pools. Finally, 122 candidate SNPs were obtained on chromosome 3. Due to genetic linkage, trait-associated differential SNPs co-segregate with their flanking variants. Using these markers, we preliminarily delimited the candidate genomic interval for the target gene to the physical position 36,471,924–36,531,664 bp on chromosome 3 (Figure 4).

The SNP-index was mapped according to its distribution on chromosomes (3–12). Calculating the average SNP-index per 1 Mb can reflect the SNP-index distribution of the two generations. Calculate △ (SNP-index), △ (SNP-index) = SNP-index (cucumber sprout)–SNP-index (cucumber sprout not). Select the window larger than the threshold at the 95% confidence level as the candidate interval. the candidate interval is located on chromosome 3. For 122 candidate polymorphism marker sites (Table 1), ANNOVAR software (version 20200607) was used to annotate and extract the results. the genes at the sites causing stop loss or stop gain, non-synonymous mutations or variable splicing were selected as candidate genes (Table 1 and Table S3).

### 2.5. Fine Localization and Identification of Target Genes

**Figure 5:**
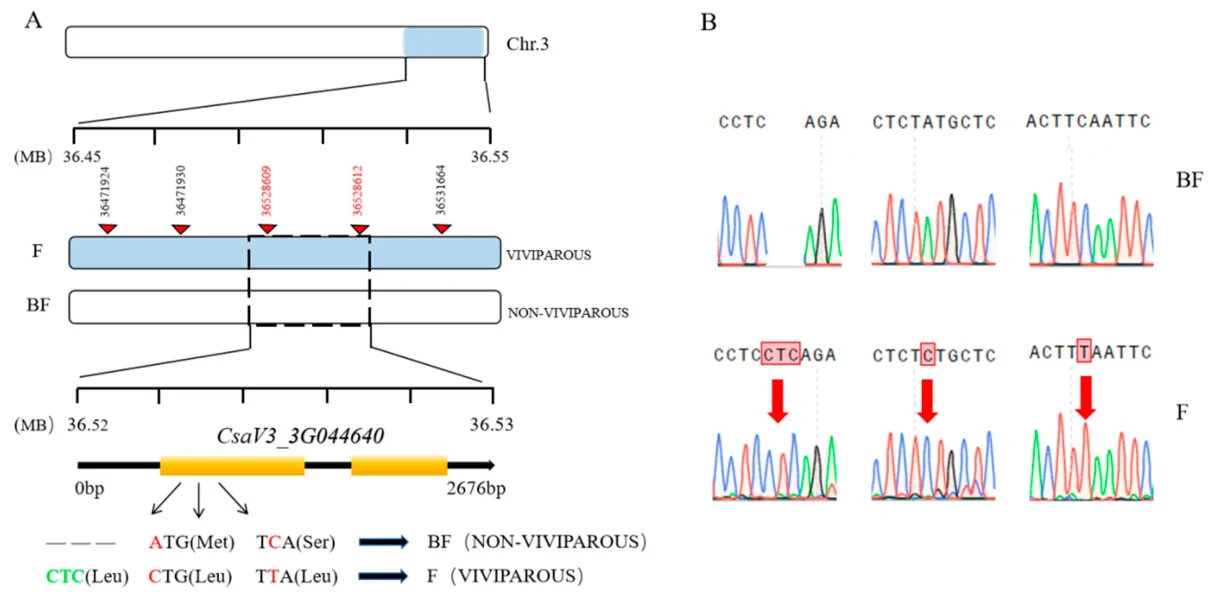
*CsCYP82D47* gene mapping and cloning: (**A**) genotyping of F_2_ individuals was performed using KASP technology. The blue bar is homozygous for the F allele and the yellow represents the exon. (**B**). Confirmatory analysis of mutation site by PCR sequencing.

To further validate and narrow the candidate interval obtained from BSA-seq, an F segregating population derived from the cross between the viviparous line F and non-viviparous line BF was constructed. Based on the preliminary mapping interval on chromosome 3, six polymorphic SNP markers were developed for fine genotyping. All F individuals were genotyped using the KASP assay, and a total of 47 recombinant plants were identified. Subsequent recombinant screening and linkage analysis further delimited the target interval to two tightly linked SNPs (SNP36528609 and SNP36528612) on chromosome 3 (Figure 5A). The refined candidate region spanned from 36471924 bp to 36531664 bp, which effectively validated the reliability of the initial BSA-seq mapping results. According to the cucumber V3 version 9930 reference genome annotation, only one protein-coding gene (*CsaV3_3G044640*) is located within this interval. This gene encodes a cytochrome P450 family protein, designated *CsCYP82D47*, which was therefore considered the key candidate gene responsible for cucumber vivipary.

### 2.6. Sequence and Cis-Component Prediction of CsCYP82D47

**Figure 6:**
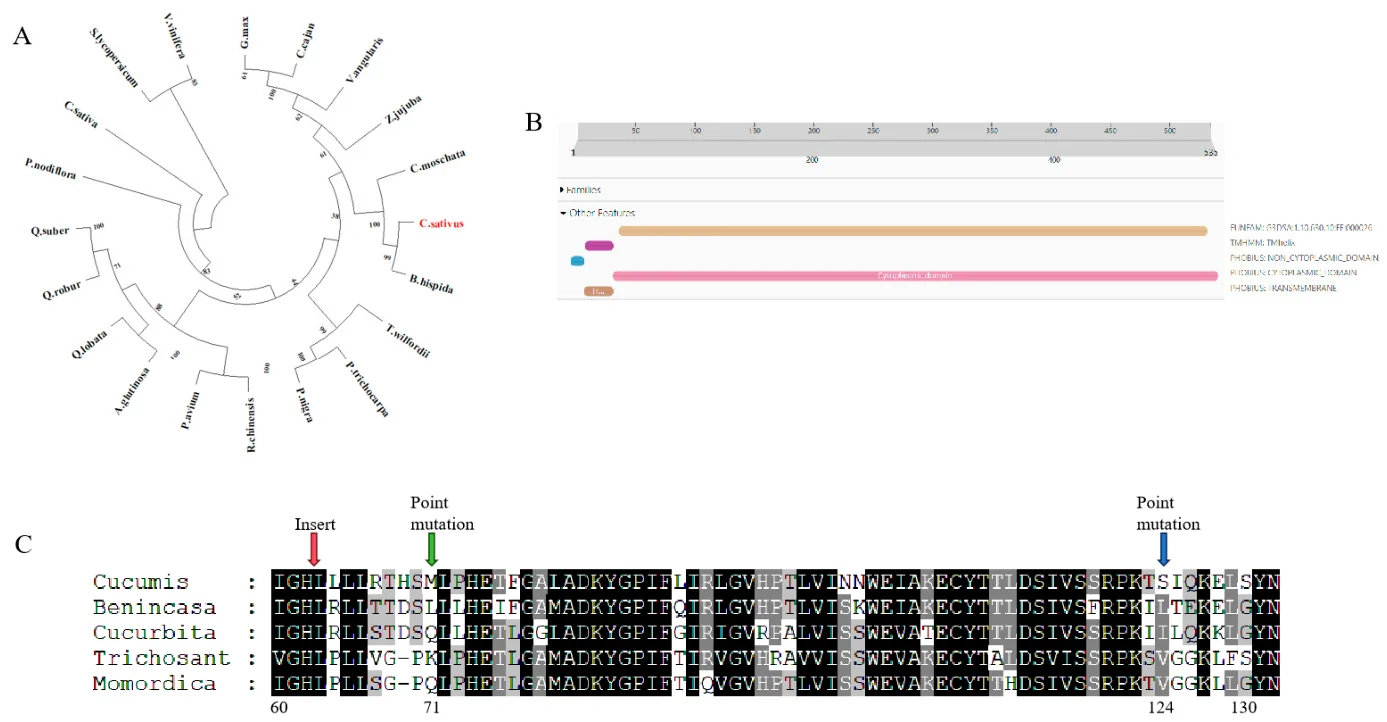
(**A**): Phylogenetic analysis of CsCYP82D47. (**B**): Domain analysis of CsCYP82D47. (**C**): Alignment of the multiple protein sequence of CsCYP82D47.

In order to validate the SNPs, the full-length of *CsCYP82D47* gene in F and BF were amplified and sequenced. Sequence comparison showed that *CsCYP82D47* had three point mutations on the first exon between F and BF. One of which had insertion of three bases (CTC, encoding leucine), among which two points mutations from base T to base C or base C to base T result in resulted in the mutation of both amino acids to leucine (Figure 5A). CsCYP82D47 is composed of 535 amino acids. Phylogenetic analysis showed that it is closest to *B. hispida* and *C. Moschata* (Figure 6A). The amino acid sequence similarity of CYP82D47 and CYP82D47 homologues from various cucurbits, including melon (*Cucumis melo*) and winter melon (*Benincasa hispida*), was high in local multiple sequence comparisons. However, for the three mutation stable points of the cucumber mutant, only the mutation inserting leucine was located in the conserved region (Figure 6C). Analysis of the structural domains of the CYP82D47 protein sequence showed that the predicted structural domains of the protein include one cytoplasmic structural domain, one non-cytoplasmic structural domain, one transmembrane structural domain and one transmembrane helical structural domain. The insertion of leucine occurred in the cytoplasmic domain (Figure 6B).

**Figure 7:**
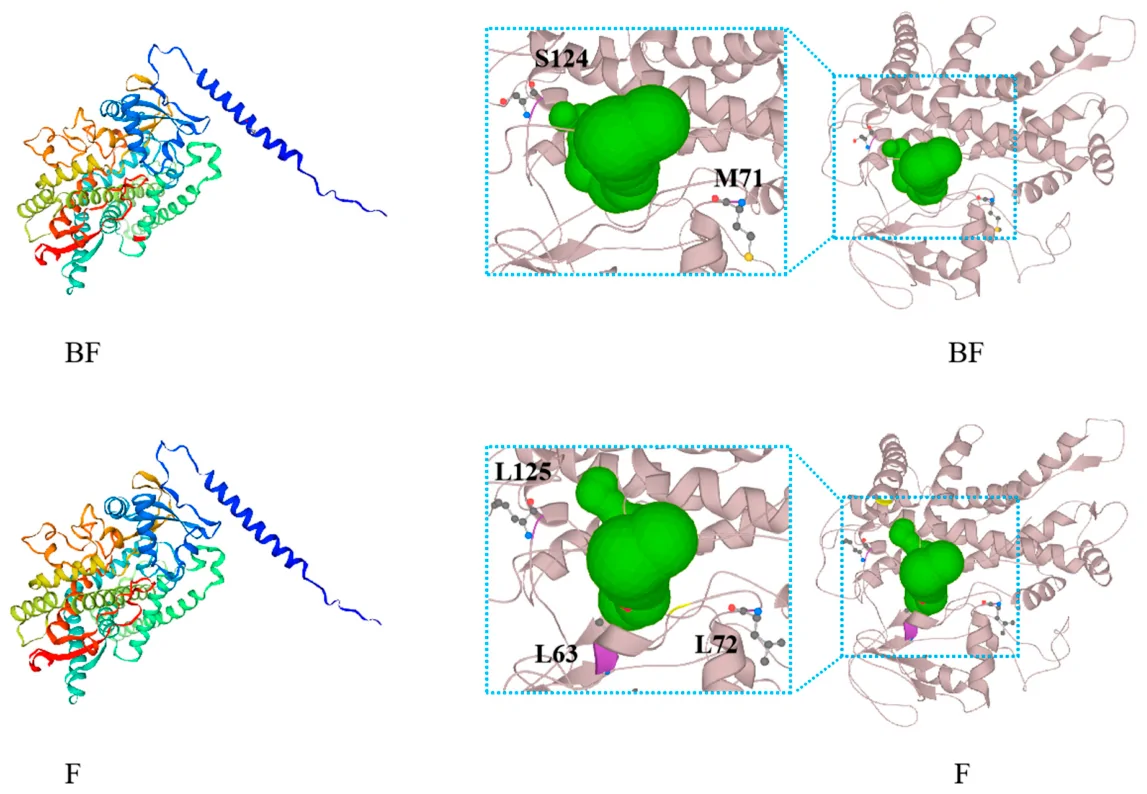
Molecular docking of CYP82D47 with tryptophan in viviparous (F) and non-viviparous (BF) cucumber. Note:The proteins are colored in rainbow gradient from the N-terminus (blue) to the C-terminus (red).

Next, the protein structure of CYP was predicted. The results showed that the three-dimensional spatial structure of the CYP mutant (F) was not significantly different from that of CYP from WT (BF) (Figure 7). In contrast, the substrate channel of CYP, which connects the active centre to the outside world, was altered by the mutation, and the bottleneck radius and length of the channel were increased by 0.5-fold and 0.3-fold (Table 2), respectively.

### 2.7. Expression Characteristic Analysis of CsCYP82D47

**Figure 8:**
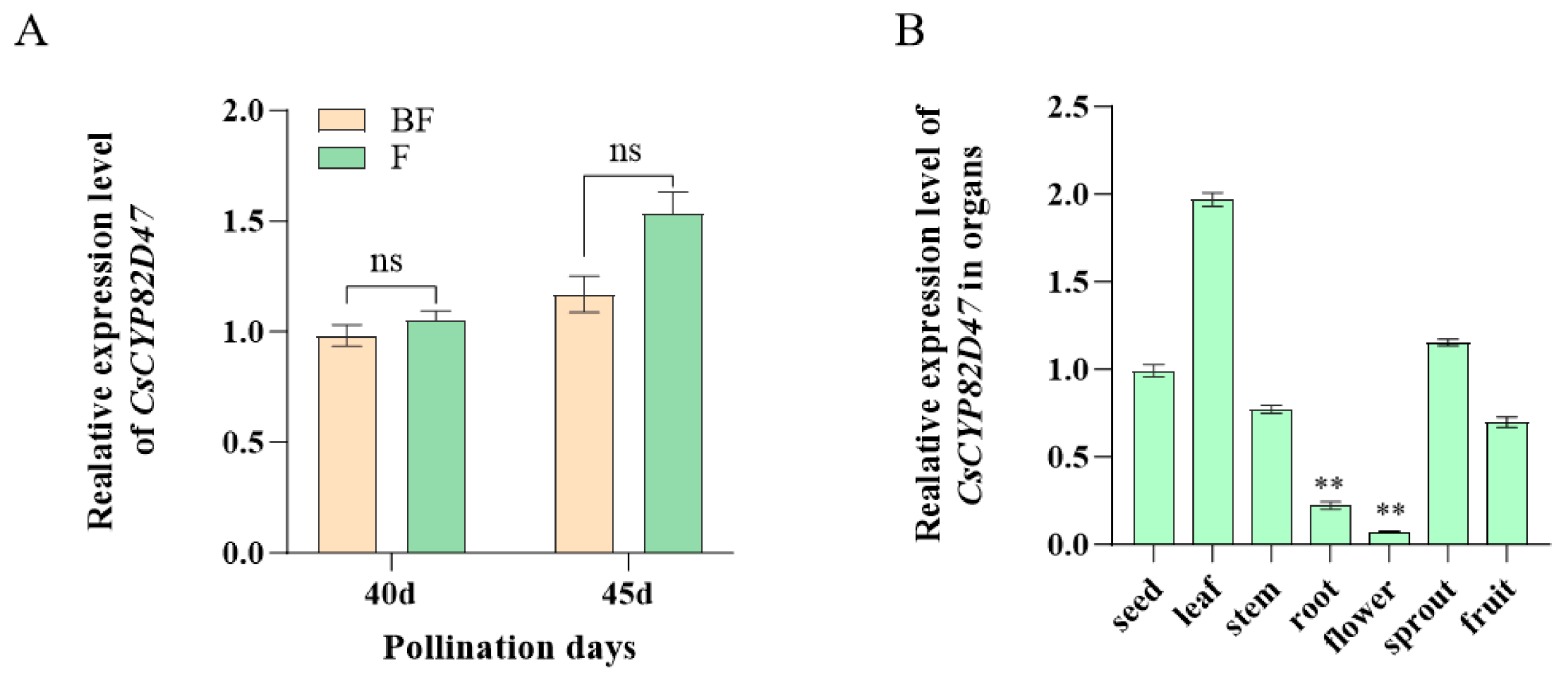
Gene structure and expression analysis of *CsCYP82D47*. (**A**): The relative quantitative expression analysis of *CsCYP82D47* in cucumber seed of F and BF. Bars are means of three replicates ± SEM. two asterisk, *p* < 0.05; (**B**): relative expression levels of *CsCYP82D47* in cucumber seed of F different tissues. Bars are means of three replicates ± SEM. two asterisk, *p* < 0.05. ns = no significant difference.

We also studied the expression profiles of *CsCYP82D47* in F and BF at different periods (Figure 8A). The results showed that the expression of *CsCYP82D47* was slightly higher in BF than F at 40 days after pollination, and the expression of *CsCYP82D47* in the non-viviparous material BF was 1.2 times that of the viviparous material F. However, after 45 days of pollination, the expression of *CsCYP82D47* in the viviparous material F was 1.6 times that of the non-viviparous material BF. In general, the expression of *CsCYP82D47* in BF materials was very low at 40 DAA. In both F and BF, the expression levels of *CsCYP82D47* were increased slowly over time. In order to understand the spatial expression characteristics of *CsCYP82D47*, we detected the expression levels of *CsCYP82D47* in F fruits, roots, stems, leaves, flowers seed and sprout (Figure 8B). The results showed that CsCYP82D47 had tissue-specific expression, and the expression of *CSCYP82D47* in leaves, sprouts and seeds was significantly higher than other tissues.

## 3. Discussion

Cucumber seeds in the development of maturity without dehydration and drying and direct germination in the parent plant, that is, the phenomenon of viviparity, will lead to seed production and quality reduction, greatly affecting the promotion of high-quality cucumber varieties. This phenomenon is also common in rice, tomato, and other crops. Researchers have systematically studied the viviparous phenomenon. In Arabidopsis, ATHB2 mediates seed dormancy and germination [31], and the IQD gene SUN24 also mediates the synthesis of ABA involved in seed germination in tomato [32]. In cucumber, there are very few studies on viviparity, and in 2017, Gao et al. initially identified *Csa4G622760* and *Csa4G622800* as viviparity-associated candidate genes by gene targeting [13]. In this study, we developed cucumber materials BF (non-viviparous) and F (viviparous) with stably inherited viviparous traits after years of cultivation. In this case, the viviparous material gradually started to sprout 38 days after pollination, and the sprouting outbreak period started at 42 days and lasted until 50 h after pollination when the sprouting rate reached 87.8%. Seeds were selected from 34 to 40 days after pollination for multiple hormone assays, in which the A hormones that promoted seed germination in F were significantly higher than those in BF (except for salicylic acid at 34 d), and the B hormones that inhibited seed germination were lower than those in BF. Paraffin sections further confirmed that the F group gradually germinated at 40 d after pollination. Based on this, we determined the stability of this cucumber material and investigated it more thoroughly.

BSA-Seq and QTL analysis is a genetic research method used to locate and quantify the contribution of those genes or loci controlling quantitative traits. BSA-Seq analysis allows the identification of chromosomal regions affecting a specific trait by linking the phenotypic variation in that trait to the genotypes of genetic markers (e.g., SNPs or microsatellites) [10]. It can be used for crop improvement, analysis of disease mechanisms, and basic genetic studies of complex traits, such as rice [33]. Here we used QTL analysis for the above F and BF groups after genetic analysis of cucumber viviparous trait, and identified *CsaV3_3G044640* as a potential cucumber viviparous candidate gene encoding the CYP82D47 protein of the cytochrome P450 family by BSA. Further identification revealed an amino acid insertion (L63) and amino acid site mutations (M71 to L and S124 to L) in the CYP82D47 protein in the viviparity lines (Figure 5).

Cytochrome P450 is a class of hemoglobin-like enzymes widely found in microorganisms, plants, and animals, which are involved in the metabolism of endogenous and exogenous substances, including drugs and environmental compounds. The substrate channel of P450 is one of the key factors affecting its catalytic properties, and the performance of the substrate channel determines how the substrate and the product move in and out of the active centre of the enzyme [34]. The substrate channels of P450 enzymes are usually located near the active centre of the enzyme, through which the substrate enters the active centre for metabolism and then the product leaves the active centre [35]. In recent years, the study of P450 enzyme substrate channels has gained increasing attention. Through computer simulation methods, it is possible to explore the static and dynamic effects of the loop in P450 enzymes on the substrate channels and reveal the molecular mechanism between the loop surrounding the entrance of the substrate channel and the substrate channel [36]. Through the loop engineering strategy, the transport properties of the P450 channel can be improved, and the opening time of the substrate channel can be enhanced, thus improving the catalytic efficiency of the enzyme.

CYP82D47 is a member of the P450 family. Previously, not much has been reported about CYP82D47. CYP82D47 was identified and characterized in sweet potato, demonstrating its regulation of total carotenoids, lutein, and others [37]. And CYP82D47 in pumpkin, bitter melon, etc., have been cloned, but their functions have not been explored. Not long ago, cucumber CYP82D47 was cloned and demonstrated that its expression was triggered by salicylic acid and ethylene glycol and involved in cucumber resistance to powdery mildew and Fusarium acnes [38]. This study preliminarily hypothesizes that cucumber CsCYP82D47 may potentially mediate the biosynthesis of germination-promoting hormones including IAA. We detected significantly higher IAA levels in viviparous line F relative to non-viviparous BF, whereas CsCYP82D47 transcript abundance showed no statistical difference between the two genotypes. This decoupling of gene transcription and hormone phenotype indicates that vivipary divergence may arise from protein structural alterations rather than transcriptional regulation. To test this hypothesis, we performed comparative computer simulations of the three-dimensional conformation and static substrate channels of CsCYP82D47 from F and BF lines, drawing on conserved structural features of the P450 family. It has been explicitly demonstrated that the channel bottleneck is critical for substrate selectivity, whereas the hydrophobicity and bulkiness of the channel-lining residues exert steric restrictions [39]. The structural modelling revealed no global conformational divergence between the two variants; nevertheless, the mutant CsCYP82D47 from F exhibited markedly extended substrate channels and enlarged bottleneck radii, which may facilitate substrate access and boost catalytic potential. Published work has documented that numerous plant P450 proteins shuttle tryptophan to active centres via substrate channels to generate IAOx, the critical biosynthetic precursor of IAA. We thus tentatively infer that CsCYP82D47 may modulate IAA homeostasis through the indole metabolic branch, and sequence mutations in viviparous cucumber may strengthen its catalytic activity toward this pathway, driving elevated IAA accumulation and triggering premature seed germination.

Notably, the regulatory mode of CsCYP82D47 differs fundamentally from previously characterized cucumber seed dormancy regulators such as CsBPC and CsABI3 [25]. The cucumber CsBPC transcription factor regulates seed dormancy and germination by repressing CsABI3 expression, revealing a canonical transcriptional regulatory cascade controlling vivipary. CsABI3 acts as a core positive effector of ABA signalling that sustains deep seed dormancy, while CsBPC suppresses CsABI3 transcription at the transcriptional level to break dormancy. Unlike these transcription factors whose functional variation mainly stems from altered gene expression, CsCYP82D47 exhibits equivalent transcript levels in viviparous and wild-type materials. Its presumed regulatory function depends on structural remodelling induced by amino acid mutations, which reshapes substrate channel architecture and shifts hormone metabolic flux, representing a distinct post-translational regulatory route for cucumber vivipary.

Sequence conservation analysis of CsCYP82D47 revealed that the two missense mutations detected in viviparous materials are evolutionarily divergent, while the leucine insertion site is highly conserved across all tested Cucurbitaceae species and shares high homology with the corresponding locus in Arabidopsis thaliana. This conserved insertion site suggests that the substrate channel-dependent regulatory pathway mediated by CsCYP82D47 may be evolutionarily conserved among plants. In conclusion, this study identifies CsCYP82D47 as a promising candidate gene linked to cucumber vivipary and proposes a putative structural hormone regulatory model distinct from the CsBPC-CsABI3 transcriptional cascade. Comprehensive biochemical assays and genetic functional validation are still required to fully resolve the complete molecular network governing cucumber preharvest sprouting.

## 4. Materials and Methods

### 4.1. Plant Materials and Field Treatment

The cucumber varieties, high generation F (viviparous) and BF (non-viviparous) material used for the trials were donated by Prof. Huiming Chen from Hunan Academy of Agricultural Sciences, Hunan Vegetable Research Institute. After multiple self-breeding of *Cucumis satirus* L., the viviparous mutant (F) was obtained from the offspring of Cs10A, a natural mutant whose characteristics showed stable heritability. F and BF cucumber seeds with full and undamaged seeds were soaked in warm water at 60 °C for half an hour, then placed at 28 °C for germination for 24 h until the seeds were exposed to white. Germination was conducted in a constant-temperature incubator (LRH-250, Shanghai Yiheng Scientific Instrument Co., Ltd., Shanghai, China).The exposed seeds were sown in the substrate and placed in an indoor greenhouse with a daytime temperature of 25 °C and a night temperature of 22 °C for seedling cultivation. The photoperiod of the greenhouse was 16/8 h. Light condition was 250μmo·m^−2^·s^−1^, and relative humidity was 70~90%. Seedlings were cultivated to two-leaf stage. And in the spring of 2021 and the spring of 2022, they were transplanted to the plastic greenhouse of Hunan Agricultural University for planting: the row width is 65 cm, and the plant distance is 30 cm. Conventional field management was conducted.

### 4.2. Field Data Statistics and Planting and Sample Collection

In the summer of 2021, after the fruit seeds were mature (about 30d after flowering and pollination), the melon dissection observation was carried out continuously. A total of 10 individual plants of each line were randomly selected for germination rate statistics, and anatomical statistics of cucumbers were performed every 2d until 50 days of pollination (germination rate reached more than 95%). We randomly divided the harvested samples into 3 parts, treated them with liquid nitrogen, and then placed them at −80 °C for storage.

Calculation formula: germination rate (%) = number of seeds/total number of seeds ×100.

### 4.3. Construction of Genetic Population

Using the high generation of cucumber inbred lines F (viviparous) and BF (non- viviparous) as parents, the cross-back combination (F × BF, BF × F) was combined in May 2022 to construct the F_1_ generation. The harvested F_1_ generation was multiplied in October 2022, and the F_1_ generation population was pollinated by single plants to make them self-cross, so as to construct an F_2_ generation isolated population containing 323 single plants.

### 4.4. Resequencing and BSA Mixed Pool Analysis

Thirty extreme viviparous and thirty extreme non-viviparous individual plants were selected from the F_2_ population to construct two mixed phenotypic pools, together with two parental lines for whole-genome resequencing (30× depth for mixed pools, 20× depth for parents).

Raw reads were quality-filtered to remove adapter-contaminated reads, reads with >10% ambiguous N bases, and low-quality reads where more than 50% of bases had Phred quality score < 20. Clean reads were aligned to the cucumber reference genome (Chinese Long v3, http://cucurbitgenomics.org/organism/20) using BWA-MEM (version 0.7.17). After marking PCR duplicates with Picard, SNP and InDel variant calling was performed using GATK (version 4.2.6.1) following the GATK best-practices workflow. Variants were further filtered with the following criteria: QD < 2.0, FS > 60.0, MQ < 40.0 were excluded. Variant annotation was completed via ANNOVAR software (version 20200607). The SNP-index of each locus was calculated, and a 1 Mb sliding window with 100 kb step length was applied to compute the averaged SNP-index within each window. The ΔSNP-index was calculated as follows: ΔSNP-index = SNP-index (viviparous pool) − SNP-index (non-viviparous pool). Permutation-based 95% confidence interval was adopted as the threshold; genomic regions where window-averaged ΔSNP-index exceeded this 95% confidence interval threshold were regarded as candidate QTL intervals.

### 4.5. Field Sampling

Female flowers blooming at node 10 of cucumber plants with consistent growth were selected for artificial pollination and tagged, and the pollination date was recorded. Cucumber seeds at 34, 36, 38, 40 and 45 days after pollination (DAP) were collected for sampling. Fresh seed samples were immediately frozen in liquid nitrogen after harvest. Seeds from independent individual plants were collected to form biological replicates rather than simply splitting homogenized samples into aliquots. All fruit samples were made in 3 copies, ground into powder and stored in an ultra-low temperature refrigerator at −80 °C.

### 4.6. Paraffin Section

Seeds of 40-day-old viviparous cucumbers and non-viviparous cucumbers were extracted and fixed in a 50% ethanol solution, then gradient dehydration was completed with 70% ethanol, −85% ethanol, −95% ethanol and anhydrous ethanol, and finally made clear with xylene and sealed with paraffin wax. The samples were cut into 10 μm thin layers, stained with a solid green dye, and recorded and examined with Olympus BX 53 biological microscope [40].

### 4.7. Determination of Plant Hormone Content

Seeds were harvested from open-pollinated viviparous and non-viviparous cucumber fruits at 34, 36, 38, and 40 days after pollination. For each cucumber genotype and each developmental stage, independent samples were collected from nine healthy individual plants, which were randomly divided into three groups to form three strictly independent biological replicates; each biological replicate contained mixed seeds from multiple fruits to eliminate individual differences. All sample processing and instrumental detection were performed with three technical replicates for each biological replicate. All fresh seed samples were immediately frozen in liquid nitrogen and fully ground into uniform powder. Subsequently, 0.2 g of each powdered sample was transferred into a 2 mL centrifuge tube, and 1.5 mL of 80% (*v*/*v*) methanol aqueous solution was added for hormone extraction. Stable isotope internal standards (200 µL of ^2^H_6_-ABA and ^2^H_5_-IAA) were supplemented into the extraction system, and the mixture was incubated at 4 °C for 12 h with ultrasonic vibration. After incubation, the samples were centrifuged at 1000× *g* for 10 min at 4 °C, and the supernatant was carefully collected. The obtained supernatant was concentrated and dried using a vacuum concentrator. For secondary extraction, 200 µL of 80% (*v*/*v*) methanol was added to the dried residue, and the mixture was incubated at 4 °C for 4 h. The supernatant was collected again and concentrated to dryness following the same procedure. After two rounds of extraction and concentration, the dried residues were redissolved in 100 µL of 10% (*v*/*v*) methanol solution, and the resulting samples were used for endogenous hormone detection via an LC-MS/MS tandem mass spectrometer [41,42,43]. All hormone content data were analyzed by one-way ANOVA using SPSS 26.0 software. The significance threshold was set at *p* < 0.05. Multiple comparisons were performed using Duncan’s new multiple range test. All data were expressed as mean ± standard error (SEM) of three biological replicates.

### 4.8. Domain and Protein Structure of CYP Prediction

We used the InterProScan software, version 5.64 (http://www.ebi.ac.uk/interpro/ (accessed on 10 June 2025)) accessed on online analysis software to predict the protein domain. The protein structure of CYP were analyzed by using the AlphaFold3 (AF3) software, version 3.0.1 (https://alphafoldserver.com/ (accessed on 10 June 2025)) online analysis software [44].

Due to access limitations of the overseas server, all structural simulation analyses were completed based on offline prediction data and published P450 structural analysis standards.

### 4.9. Real-Time Fluorescent Quantitative PCR

Fresh seeds were harvested from fruit flesh of both BF and F at two developmental time points (40 DAP and 45 DAP, where DAP stands for days after pollination). In addition, tissue samples from different organs of F were collected. Each sample type included three independent biological replicates; each biological replicate contained mixed tissues pooled from multiple individual plants. Total RNA was extracted using TRIzol reagent. RNA integrity and purity were assessed by agarose gel electrophoresis and NanoDrop spectrophotometry. Genomic DNA contamination was removed by DNase-I treatment before reverse transcription. A fixed amount of total RNA (1 μg) was used for first-strand cDNA synthesis with a commercial reverse-transcription kit. qRT-PCR was performed with the SYBR Premix Ex-Taq™ II kit (Takara Biomedical Technology Co., Ltd., Beijing, China) on an iQ™5 Multicolor Real-Time PCR Detection System (Bio-Rad Laboratories, Hercules, CA, USA) [45]. Primer amplification efficiency was evaluated before quantitative analysis. The cucumber UBQ gene was used as the internal reference gene, and relative gene expression levels were calculated via the 2^−ΔΔCt^ method. Each biological replicate was subjected to three technical replicates for qRT-PCR amplification. Statistical analysis of gene expression differences was performed using SPSS 26.0 software, and significant differences were determined by independent-sample t-test at *p* < 0.05. Quantitative primers for UBQ, CsSUN, and CaMs/CMLs genes are shown in Table S4.

## 5. Conclusions

Owing to the environmental lability of cucumber vivipary and the scarcity of near-isogenic line resources, the molecular basis of cucumber vivipary remains poorly characterized. Here, we performed multi-generation phenotypic, cytological, and genetic analyses using two laboratory-developed cucumber lines, non-viviparous BF and viviparous F. Vivipary was initiated at the late seed maturation stage, characterized by radicle penetration of the seed coat. Seed endogenous phytohormones of both genotypes displayed dynamic changes during fruit development. At 40 days after pollination (DAP), line F exhibited typical vivipary, while BF seeds remained dormant. At this stage, F seeds accumulated significantly higher levels of salicylic acid, gibberellin 1, gibberellin 3, 3-indoleacetic acid (IAA), and jasmonic acid, but lower abscisic acid content, compared with BF. Genetic analysis indicated that cucumber vivipary is likely regulated by a single major-effect gene.

Combining BSA-seq and QTL mapping, we localized the major vivipary-associated locus and identified *CsaV3_3G044640*, which encodes the cytochrome P450 protein CsCYP82D47, as the primary candidate gene. Transcript analysis revealed that CsCYP82D47 exhibits obvious tissue-specific expression and is highly expressed in leaves, sprouts, and seeds. However, its expression level did not differ significantly between viviparous and non-viviparous lines. Sequence alignment identified multiple allelic variations in viviparous materials, including one amino acid insertion (L63) and two missense mutations (M71L and S124L) in the CsCYP82D47 protein.

Structural simulation suggested that these leucine-related substitutions expand the substrate-binding channel and enhance the catalytic capacity of mutated CsCYP82D47, which may increase endogenous IAA accumulation and induce seed vivipary. Importantly, this regulatory model linking CsCYP82D47 structural variation to hormonal alteration and the vivipary phenotype represents a plausible hypothesis. The current inference is supported by indirect evidence from genetic mapping, hormone profiling, and structural modelling; direct functional validation is still required to confirm this mechanism in future studies.

In conclusion, this study identifies a key candidate gene for cucumber vivipary. Our results provide a fundamental basis for uncovering the molecular mechanism of cucumber vivipary and facilitate the future application of this gene in cucumber molecular breeding.

## Figures and Tables

**Table 1:** Annotation of variation sites.

Category		Number of SNPs
Upstream		21
Exonic	Stop gain	0
	Stop loss	0
	synonymous	4
	non-synonymous	2
Intronic		22
Splicing		0
Downstream		13
Upstream/Downstream		5
Intergenic		52
ts		62
tv		60
ts/tv		1033
Total		122

**Table 2:** The static tunnel analysis of P450CYP BF and F.

CYP82D47	Tunnel Length (Å)	Bottleneck Radius (Å)
BF	31.5	1.2
F	40.6	1.8

## Data Availability

The original contributions presented in this study are available within the article and its Supplementary Materials. Further inquiries can be addressed to the corresponding author.

## Supplementary Materials

The following supporting information can be downloaded at https://www.mdpi.com/article/10.3390/ijms27167428/s1.
