# Supplementary material for: CsCYP82D47 Is Identified as a Candidate Gene for Vivipary in Cucumber (Cucumis sativus L.)
Source: Int J Mol Sci. 2026 Aug 19;27(16):7428. doi: 10.3390/ijms27167428 (PMC13512908; doi:10.3390/ijms27167428)
Supplement: Supplementary file 1 [file ijms-27-07428-s001.zip › ijms-4437955-supplementary.pdf]

Supplement S1

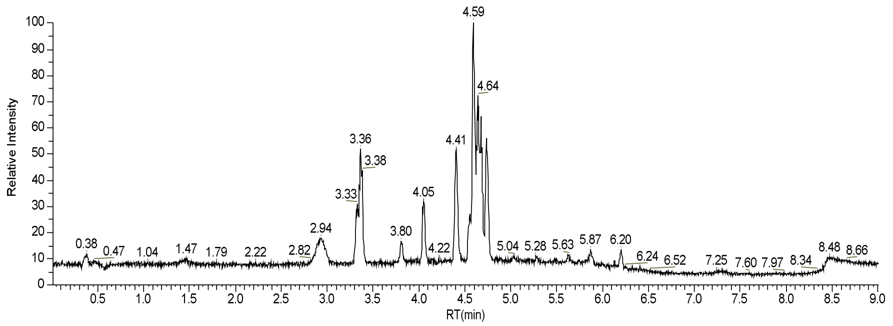

Total Ion Flow Diagram for Standards

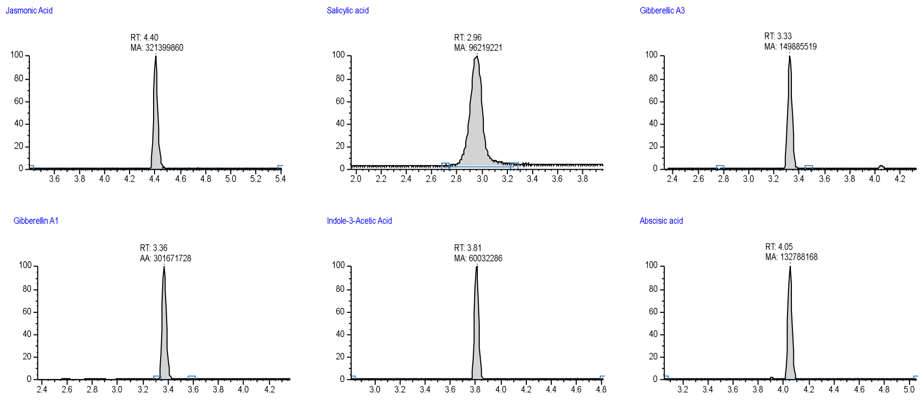

Standard extraction ion flow diagram

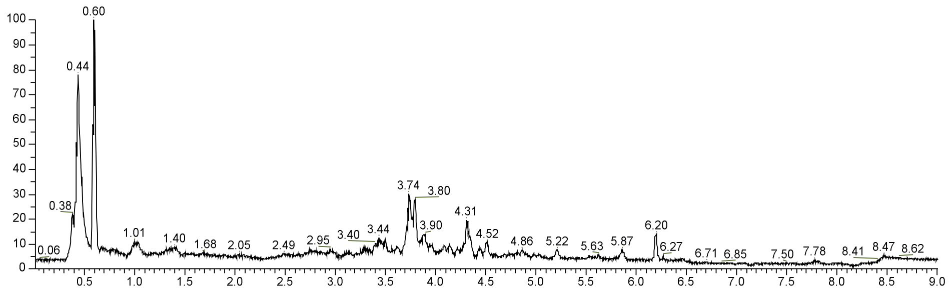

Sample total ion flow diagram

Supplement S2

Table S1 Percentage of average viviparity of P1, P2, F1 and F2population plants.

Generation	Sample number	percentage of average viviparity （%）	Range（%）	CV（%）
P1	18	50.51±15.22a	26.57~76.36	30.13
P2	18	0c	0.00~0.00	0
F1	18	22.65±8.21b	10.32~33.21	36.25
F2	323	23.11±20.71b	0.00~96.65	89.62

Table S2 Frequency distribution of viviparity percentage.

Generation	The ratio of preharvest germination (%)											Sample number	Sample average (%)	Standard deviation(%)
	0.0	0.1~10.0	10.1~20.0	20.1~30.0	30.1~40.0	40.1~50.0	50.1~60.0	60.1~70.0	70.1~80.0	80.1~90.0	90.1~100.0			
P_1	0	-	-	1	3	6	3	3	2	-	-	18	50.51	15.22
P_2	18	-	-	-	-	-	-	-	-	-	-	18	0	0
F_1	-	-	7	6	5	-	-	-	-	-	-	18	22.65	8.21
F_2	35	61	99	33	30	28	11	10	7	5	4	323	23.11	20.71

Supplement S3

Table S3. Annotation status of candidate InDel sites

Category		Number of Indels
Upstream		2
Exonic	Stop gain	0
	Stop loss	0
	Frameshift deletion	0
	Frameshift insertion	0
	non-Frameshift deletion	0
	non-Frameshift insertion	0
Intronic		3
Splicing		0
Downstream		4
upstream/downstream		2
Intergenic		23
Insertion		21
Deletion		14
Total		35

Table S4. Primer sequence

Gene	Forward primer（5’-3’）	Reverse primer（3’-5’）
CsaV3_3G044640	ATGGTTTGTGGGAAGCGGAT	CCGTTACCTGACCTGCCAAT
CsCYP82D47	ATCATTGGGGCTTCCCACAG	CCGTTACCTGACCTGCCAAT
	ATTGGCAGGTCAGGTAACGG	CTGTCCAAACTCGAGGGTCC
	AAAGCCACTTGCCTGACACT	CAGCCATGTCCACTGGTTCA
Ef1α	CCAAGGCAAGGTACGATGAAA	AGAGATGGGAACGAAGGGGAT
